# The 4th annual American Society for Intercellular Communication (ASIC) meeting, 2024 conference report

**DOI:** 10.20517/evcna.2025.51

**Published:** 2025-09-03

**Authors:** Ashley E. Russell, Aurelio Lorico

**Affiliations:** ^1^Department of Biology, School of Science, Penn State Erie, The Behrend College, Erie, PA 16563, USA.; ^2^Department of Basic Sciences, College of Medicine, Touro University Nevada, Henderson, NV 89014, USA.

The fourth annual meeting of the **American Society for Intercellular Communication (ASIC)** was held in Baltimore, Maryland, from October 17th-19th, 2024, and featured groundbreaking findings that are pushing the field of extracellular vesicles (EVs). Highlights included the discovery of a new pathway for selective RNA loading into EVs, new evidence linking EVs to neuroinflammation, cancer progression, and immune suppression, and compelling demonstrations of their potential in brain-targeted delivery, tissue repair, and cancer immunotherapy. Collectively, these advances underscore EVs as both key drivers of disease and powerful tools for next-generation diagnostics and therapeutics.

The meeting drew over 130 participants and was supported by twelve sponsors, including Caerus, Ceres Nano, Kinetic River, Particle Metrix, Beckman Coulter Life Sciences, Alpha Nano Tech, Spectradyne, Univercells Technologies, Unchained Labs, MetvareBio, USA Scientific, and Kinetic River. The three-day program featured fifty oral presentations, an NIH Grant Writing Workshop designed for early-career investigators and trainees, and an NIH roundtable with six program officers. Additionally, a poster session was held on the evening of the second day, showcasing twenty-eight posters.

**Day 1 of the Meeting:** The meeting opened with remarks from the current president of ASIC, Fatah Kashanchi (George Mason University). The first session of the day was an NIH workshop/Pre-Program session, moderated by Shilpa Buch (University of Nebraska) and Theresa Whiteside (University of Pittsburgh).

The first presentation was given by Christine Happel (NIH/NCATS), co-leader of the extracellular RNA (exRNA) communication program. This program has resulted in 79,000 citations and has seen a consistent increase in publications during the last 3 years. Two-thirds of R01 grants were awarded through unsolicited funding opportunities, with the most frequent study section being Molecular Genetics. Most applications were reviewed by Special Emphasis panels. The increase in SBIR/STTR grants suggests that exRNA studies are increasingly moving toward commercial applications.

The second talk was given by Nichole Darringer, who provided an overview of the funding mechanisms offered by the National Institute of Biomedical Imaging and Bioengineering (NIBIB). NIBIB coordinates imaging and engineering research with other NIH institutes and agencies to facilitate the translation of technologies into medical applications. She highlighted three requirements for NIBIB applications: technical development, broad platform technology, and biomedical application. No clinical trials are required. Besides R01 and R03 grants, she recommended high-risk, high-reward mechanisms, as well as K01, F32, and K99 opportunities for trainees.

The third and last NIH talk was delivered by Konstantin Salnikow (NIH/NCI), Program Director of the Division of Cancer Biology. He discussed the NCI’s mission to support cancer research with health-centric and scientifically empowering goals. In 2023, NCI funded 5,700 research projects in basic cancer research, with an additional objective of training a diverse workforce to advance the field. Dr. Salnikow noted that NCI is the only NIH institute whose Director can submit the budget directly to the US President. The most recent NCI budget was $7.2 billion, with 18% allocated to intramural research, 74% to extramural programs, and the remainder for research management support. The current payline for all applications is 12%.

For the first time, ASIC included a review session on the EV field. The session opened with presentations by Shilpa Buch (University of Nebraska) and Julie Saugstad (Oregon Health and Science University), who presented an overview of the current literature on EVs in the central nervous system. Shilpa Buch emphasized the critical role of glial cells during the asymptomatic phase of many neurodegenerative diseases. She then discussed how EVs carry misfolded proteins, highlighting the diagnostic potential of phospho-tau and alpha-synuclein in tauopathies, including Alzheimer’s and Parkinson’s disease. A recent finding that EVs are more present in urine than in plasma suggests that urine may be a preferable medium for developing EV-based diagnostics for neurodegenerative diseases. The presentation also reviewed the types of miRNAs and long non-coding RNAs carried by EVs and their links to neurodegenerative diseases. Julie Saugstad then discussed the methods for isolating EVs from cerebrospinal fluid, noting challenges such as the limited sample volume and biological heterogeneity. She also discussed the therapeutic EVs containing long non-coding RNAs for neurodegenerative diseases.

Lucia Languino (Thomas Jefferson University) presented an overview of EVs in cancer research. Over the last 22 years, more than 15,000 cancer-related peer-reviewed papers on EVs have been published, with the majority appearing in recent years, reflecting the field’s increasing scientific relevance. As the field originated in Europe, the US is currently seeking to enhance its competitiveness. Languino described how cancer-derived EVs and extracellular non-membranous particles (collectively EVP) promote tumor growth, motility, epithelial-mesenchymal transition, angiogenesis, and pre-metastatic niche formation. She also discussed current challenges in using EVs as cancer therapeutics, including side effects, immunogenicity, low efficacy due to drug resistance, limited access to cellular/molecular targets, complexity of the biological systems, and inter-individual variation. Benefits included low immunogenicity, especially when using autologous EVs, the ability to modify EVs to improve therapeutic efficacy, and their capacity to infiltrate tissues and cross the blood-brain barrier. She also discussed EVs as an important diagnostic tool for liquid biopsy, noting that cargo signatures from primary tumors may differ from those of metastases. Languino concluded with recommendations to address gaps in knowledge, including large-scale clinical sample screening, preferring plasma over serum to reduce platelet contamination, analyzing EV “coronas,” and studying cancer stem cell-derived EVs.

Olivier Loudig (Hackensack Meridian Health) and Daniel Chiu (University of Washington) presented on EVs in diagnostics. Loudig emphasized that EVs protect their cargo (proteins, lipids, RNA, and small DNA fragments) better than free circulating nucleic acids. EVs can be isolated from all body fluids, including blood, CSF, saliva, urine, milk, lymph, bronchial lavage, and synovial fluid. Recently, breath condensate has been identified as a source of EVs for lung disease diagnostics. miRNAs are particularly valuable as EV biomarkers because they often remain intact, unlike mRNAs. In addition to EVs, the recent discovery of exomeres and supermeres has expanded the diagnostic potential of EVP. Chiu discussed commonly used methodologies for diagnostic pipelines, including size-exclusion chromatography, density gradients, and immune-isolation, as well as current knowledge gaps, including the impact of patient demographics and medical history on EV-based diagnostics.

Fatah Kashanchi (George Mason University) reviewed EVs in infectious diseases. Strikingly, the ratio of EVs containing viral products and complete virions is around 99:1, because EVs are always released along with viruses. Kashanchi explained Pathogen-Associated Molecular Patterns (PAMPs) and Damage-Associated Molecular Patterns (DAMPs). He suggested that inhibitors of EV release or uptake may serve as therapeutics for various infectious diseases. He also highlighted the role of autophagy, noting that virus-induced blockade of the autophagy pathway increases the extracellular release of EV proteins that cannot be degraded. A major knowledge gap is the current inability to efficiently separate EVs from virions and the need to study EVs in non-cancerous or non-transformed cells in infection research.

The session concluded with a presentation by Elena Batrakova (George Mason University) and Heather Branscome (American Tissue Culture Collection) on EVs in therapeutics. They highlighted the higher therapeutic potential of stem cell-derived EVs compared to stem cells themselves for regenerative applications. They discussed loading stem cell-derived EVs with drugs or engineering them with DNA or RNA molecules. These EVs may display immunomodulatory/anti-inflammatory, anti-fibrotic, anti-apoptotic, pro-migratory, and antioxidant effects. New EV therapeutic approaches include using CRISPR/Cas9 or proteins to selectively incorporate molecules into EVs. Knowledge gaps include scalable and standardized manufacturing platforms, enhanced purification techniques to decrease heterogeneity, increased stability and shelf-life, batch-to-batch consistency, regulatory compliance, optimal EV dosing, and understanding effects on target vs. non-target cells.

The Junior Faculty Session was moderated by Mỹ Mahoney (Thomas Jefferson University) and Louise Laurent (University of California San Diego). It began with a presentation by Suvendra Bhattacharyya (University of Nebraska) on miRNA export from mammalian cells, focusing in particular on the role of Syntaxin-5 (STX-5) in the reversibility of miRNA-mediated repression. Regulated miRNA export is essential for maintaining gene expression homeostasis. STX-5 prevents lysosomal targeting of multivesicular bodies (MVB) and regulates their fusion with the apical plasma membrane. By increasing the export of EV-associated miRNA, STX-5 decreases intracellular miRNA levels, thereby promoting the expression of genes otherwise repressed by miRNAs. Beyond its role in MVB fusion, the N-terminal disordered region of STX-5 can directly bind miRNAs. Through these mechanisms, STX-5 induces inflammatory responses in pathogen-infected macrophages. The therapeutic potential of STX-5-engineered EVs remains to be further explored.

Lillian Skeiky (Uniformed Services University) presented a talk entitled “Assessing the relationship between pro-inflammatory cytokines and sleep physiology following total sleep deprivation in plasma and extracellular vesicles”. Sleep loss is known to alter immune-related pathways, including cytokine production, antibody response, and T cell-mediated immunity. In patients with traumatic brain injury, poor sleep is associated with elevated levels of pro-inflammatory cytokines in EVs. In a clinical trial, 25 adults underwent 38 hours of total sleep deprivation at home, followed by 12 hours of recovery sleep, with blood samples collected after both sleep deprivation and recovery. Multiplex pro-inflammatory cytokine assays revealed that interleukin-1 levels in both plasma and EVs were associated with prolonged stage 1 sleep. Overall, pro-inflammatory cytokines in EVs and plasma appear to influence different aspects of sleep physiology. Future studies focusing on neuronal EVs will provide further insights into how sleep deprivation affects CNS-related biomarkers.

The next talk, by Yohei Nose (Icahn School of Medicine, Mt. Sinai), was entitled “Comparative Analysis Reveals No Significant Difference Between Extracellular Vesicular Particles Proteo-Transcriptome from Fresh or Frozen Tissue”. While several studies have compared fresh and frozen plasma in this regard, little is known about tissue-derived EVPs. Demonstrating that frozen tissue is a viable alternative to fresh samples in cancer research could transform EVP research. In this work, lung and prostate cancer tissues and adjacent normal tissues were enzymatically dissociated, centrifuged, and either analyzed immediately or frozen at -80 ^o^C followed by liquid nitrogen storage. Frozen samples were thawed either rapidly (10 min in pre-warmed medium) or slowly (in 1 mL medium) and compared to fresh preparations. Using inflammation protein arrays, bulk RNA sequencing, and single-cell transcriptomics, the study found no significant differences across the three preparation protocols, except in normal lung tissue. These results suggest that frozen tissue can be a valid alternative to fresh tissue in EVP cargo analysis for cancer studies.

Monique Anderson **(**Harvard University) presented the talk “Mutations in VPS37A are associated with changes in extracellular vesicle export”. VPS37A is a component of the ESCRT-I complex, which bridges ESCRT-0 and ESCRT-II in endosomal sorting and contributes to exosome formation by mediating plasma membrane invagination and recruitment of additional endosomal components. Anderson showed that VPS37A knockdown reduces exosome production, while overexpression of mutated VPS37A (mutations associated with transverse myelitis and hereditary spastic paraparesis) resulted in an ~80%-90% increase in exosome release. Overexpression of mutant VPS37A also altered the antigenic composition of exosomes. Given the therapeutic implications of modulating exosome number and cargo, these findings warrant validation using CRISPR/Cas9 models.

The following presentation, “Extracellular vesicles derived from intravenous immunoglobulin replacement therapy serving as decoys to suppress immune response,” was given by Mỹ Mahoney (Thomas Jefferson University). Intravenous or subcutaneous immunoglobulins (IVIg and SCIg), derived from pooled plasma from 1,000-100,000 healthy donors, are widely employed to boost the immune system of patients with antibody deficiencies or to suppress inflammation in various syndromes. Mahoney showed that IVIg and SCIg also contain large numbers of EVs and their corona-associated cytokines. Multiplex profiling of 48 cytokines in EVs isolated from IVIg and SCIg revealed elevated levels of the anti-inflammatory cytokine interleukin-10 and reduced levels of 20-30 pro-inflammatory cytokines compared to EVs from normal human plasma. In particular, the pro-inflammatory cytokine RANTES was reduced 1,000-fold, whereas interferon-γ was markedly increased. Notably, IVig-derived EVs blocked interferon-γ-induced JAK/STAT1 activation, consistent with the observation that IL-10 and interferon-γ can cooperatively suppress immune cell activation,

The Junior Faculty Session concluded with remarks from sponsor Kevin Dolan from Particle Metrix, who outlined the features of the ZetaView nanoparticle analysis instrument, particularly the advantages of its four-laser configuration and its ability to combine nanoparticle tracking analysis with zeta potential measurements.

**Day 1 of the meeting:** The evening session, moderated by Aurelio Lorico (Touro University Nevada) and Sabita Roy (University of Miami Health System), focused on EVs and Biogenesis.

The first talk was presented by Keynote Speaker Alissa Weaver (Vanderbilt School of Medicine), entitled “Biogenesis and characterization of RNA-containing extracellular vesicles”. RNA incorporation into EVs occurs through interactions with RNA-binding proteins (RBPs). Contact between the ER and late endosomes can facilitate the transfer of RNA-RBP complexes into EVs. Since VAP-A is a key linker protein at ER-endosome contact sites, VAP-A knockdown reduces ER-endosome interactions. VAP-A also regulates the biogenesis and small RNA content of both small and large EVs. Importantly, this effect does not reflect changes in total cellular RNA content but rather alterations in RNA sorting into EVs. Because of their heterogeneity, not all EVs contain RNA, and VAP-A specifically regulates the biogenesis of RNA-containing dense EVs. To demonstrate this, the group isolated RNA-containing dense and small EVs, as well as RNA-negative light EVs. The light fraction was enriched in Alix, syntenin, TSG101, and CD63, while the dense fraction contained higher levels of flotillin-1. VAP-A knockdown selectively affected the small and dense EV populations. Additionally, VAP-A regulated miR-100 transfer and EV ceramide content, the latter being crucial for membrane curvature. The RNA-containing dense EV fraction was also shown to be critical for the *in vivo* growth of xenograft mouse tumors. Based on these findings, the authors proposed a model in which ceramide transfer at ER contact sites drives the biogenesis of small, dense EVs containing RNA-RBP complexes.

Next, Kevin Morris (Queensland University of Technology) presented “RNA-Exosome Therapeutics to Treat Viral Infections”. EVs are relatively inert, distribute systemically, and can cross the blood-brain barrier. Moreover, EV-producing cell lines can be engineered to package virtually any therapeutic RNA, making them promising vectors for gene therapy. For instance, EVs successfully delivered a zinc finger protein-EGFP fusion to epigenetically silence HIV in the brain. *In vivo* studies showed that retro-orbital injection preferentially directed EVs to the lung, while intraperitoneal injection targeted the liver. Furthermore, shRNA-loaded EVs effectively repressed HIV and HTLV-1 in recipient cells.

This was followed by a talk from Louise Laurent (University of California San Diego) entitled “Characterization of placental exRNA carrier heterogeneity using multi-modal molecular profiling and single-vesicle analysis”. Placental EVPs play a critical role in fetoplacental-maternal communication, yet their heterogeneity and diverse cargo complicate identification of the subtypes most relevant to this process. To address this, multiple isolation and fractionation techniques, molecular profiling, and single-vesicle analyses were applied. Results showed that placental alkaline phosphatase (PLAP)-positive EVPs exhibited low levels of tetraspanins. PLAP, a membrane-bound enzyme expressed in the placenta, enters maternal circulation after the 12th week of pregnancy. EVPs smaller than 50 nm (exomeres and supermeres) lacked PLAP, while large EVPs (> 168 nm) expressed high levels. Immune isolation further revealed three distinct subpopulations of placental EVs: CD9+CD81+, PLAP+CD63+, and CD63+.

Robert Fullem (Baylor College of Medicine) then presented “ExRNA Atlas v3 map of extracellular RNA in human biofluids sheds light on the exRNA “dark matter” beyond previously annotated genomic region”. The exRNA Atlas is a publicly available resource containing extracellular small RNA-seq profiles from over 11,000 samples. Atlas 1.0 (2019) included deconvolution by carrier type, while Atlas 2.0 (2023) integrated correlation footprinting of exRBP loci. Atlas 3.0 advances further by addressing unannotated regions (“exRNA dark matter”) through the development of a Genomic Location ID registry, producing stable identifiers for exRNA loci and enabling their mappings across transcripts and reference genome assemblies.

Next, Aurelio Lorico (Touro University Nevada) gave a talk entitled “Transport of the POMC pro-hormone by blood plasma extracellular vesicles”. The study reported that ~5% of blood EVs carried POMC bound to one of its receptors, including the five melanocortin receptors and the μ-opioid receptor. Among these, melanocortin receptor 1 was the main receptor transporting POMC on circulating EVs. Treadmill exercise for 50 min resulted in a several-fold increase in POMC-bearing EVs in circulation. Since EVs can cross the blood-brain barrier, this newly identified hormonal transport mechanism may explain the central effects of β-endorphin.

The last talk of the session was delivered by Giacomo Vacca (Kinetic River) titled “Paving the Way Toward a Urine-Based Assay for Early Diagnosis of Kidney Disease”. He presented a new version of the Delaware flow cytometer, capable of high-resolution, clog-free exosome analysis using up to five lasers and six fluorescence channels. This technology enabled the detection and differentiation of polycystic kidney disease EVs in urine by identifying polycystin-1+ EVs, which represented 40% of the total membrane-stained EV population.

The evening concluded with a highly attended poster session.

**Day 2 of the meeting:** The morning session on EVs and Infection was moderated by Ramin Hakami (George Mason University) and Gerardo Kaplan (Food and Drug Administration) and featured eight talks.

The first presentation, delivered by Robert Molestina (American Tissue Culture Collection), was entitled “Role of Babesia Secreted Extracellular Vesicles in the Modulation of the Immune Response”. Babesiosis is an emerging tick-borne disease, generally asymptomatic except in immunocompromised individuals. A network of vesicular tubes extends from the plasma membrane of Babesia into the cytoplasm of red blood cells. The researchers hypothesized that EVs from infected erythrocytes induce phenotypic changes in immune cells and alter responses to the parasite. To test this, mice were immunized with Babesia-infected erythrocyte cultures and specific antibody responses were assessed. Immunization resulted in strong IgG and IgM reactivity to Babesia antigens and conferred protection against subsequent challenge with infected red blood cells. *In vitro*, Babesia-derived vesicles taken up by macrophages activated NF-kB and stimulated the production of immunomodulatory cytokines. Future work will focus on optimizing EV isolation methods to obtain highly enriched parasite-derived vesicles.

The next talk, “Bacterial Extracellular vesicles in opioid associated co-morbidities,” was given by Sabita Roy (University of Miami Health System), who presented data on the role of inflammation in morphine-induced analgesic tolerance. Morphine tolerance was attenuated in germ-free mice and reversed by gut microbiota, consistent with the concept of a microbiota-gut-brain axis where EVs from specific gut bacteria reach the central nervous system, contributing to pain and anxiety. EVs were purified from Lactobacillus (commensal) and Enterococcus (pathogen); only the latter disrupted barrier function in IEC6 cells. Roy also reported expansion of both Gram+ and Gram- bacteria in opioid-dependent, HIV-infected mice. Morphine dependence decreased commensals while increasing pathogenic bacteria. Finally, EVs derived from both Gram+ and Gram- bacteria induced NLRP3 inflammasome expression in a macrophage cell line.

This was followed by Ramin Hakami (George Mason University), who presented” Characterization of sEV-associated RNA Species that Activate Innate Immune Response during Infection with Cytoplasmic RNA Viruses”. Rift Valley fever virus (RVFV), a single-stranded RNA virus causing a zoonotic disease endemic in Africa, was employed as a model to study the innate immune response triggered by EVs released during infection (EXi-RVFV). Purified EXi-associated RNAs were found to activate IFN- via the RIG-1 pathway. Each of the three RVFV genomic and anti-genomic segments was independently capable of inducing IFN- activation via RIG-1 signaling.

Pallavi Singh (Yale University) presented “Parasite-Derived Vesicles in Pathogenesis and Immunity”. The study focused on Babesia microti and Babesia duncani, parasites that cause severe human infections. The researchers investigated EV-mediated trafficking and delivery of parasite proteins to the erythrocyte surface. They found that B. microti and B. duncani delivered the immunodominant proteins BmGPI12 and BdV38/234, respectively, to the erythrocyte surface. Targeting Babesia EV processes may represent a promising therapeutic strategy against parasitic infection.

The next talk, presented by Md Habibur Rahman (Johns Hopkins University), was entitled “Neuroinflammation drives fentanyl addiction and relapse by a mechanism that involves the release of extracellular vesicles with modified miRNA cargo from astrocytes”. Chronic opioid dependence was shown to trigger neuroinflammation by activating microglia and astrocytes, with astrocyte-derived EVs (ADEVs) influencing fentanyl self-administration in mice. Opioid exposure altered the miRNA cargo of ADEVs, particularly miRNAs linked to opioid and reward signaling pathways. Inhibition or inactivation of nSMase2 also affected opioid self-administration, suggesting that a subset of EVs contributes directly to addiction mechanisms.

Gerardo Kaplan (FDA) then presented “Production dynamics of infectious exosome and viral particle during *in vivo* and *in vitro* hepatitis A virus (HAV) infection”. HAV can be transmitted via several routes, and viral particles may exist as either naked virions or quasi-enveloped particles formed through the ESCRT pathway. The study demonstrated that these quasi-enveloped particles are in fact infectious exosomes, as they contain unencapsidated RNA within the vesicle but outside the viral capsid. ESCRT-independent, nonlytic release of naked virus was also observed, although the underlying mechanism remains to be clarified.

The following speaker, Xuesong Chen (University of North Dakota), gave a presentation entitled “Role of endolysosomes in SARS-CoV-2 spike-induced cellular senescence in astrocytes”. After SARS-CoV-2 infection, the S1 subunit of the spike protein can persist in the bloodstream for up to 12 months and cross the blood-brain barrier (BBB). It has been shown to be endocytosed by both neurons and astrocytes. In neurons, this leads to neurite dystrophy, while in astrocytes, it initiates inflammatory cascades through activation of TLR7 in endolysosomes. TLR7 activation can also induce cellular senescence, potentially contributing to neurological complications observed in long COVID.

The last speaker of this session, Ngoc Do (Spectradyne), introduced Spectradyne’s ARC Particle Analyzer, a microfluidic-based system for counting and sizing particles.

The second morning session on EVs and CNS was moderated by Ashley Russell (Penn State Erie, The Behrend College) and Catherine DeMarino (NIH/NINDS) and featured four talks.

The first speaker, Prasun Datta (Tulane University School of Medicine), presented on “Dysregulation of mitochondrial function in neurons by extracellular vesicles from HIV-1 infected macrophages”. EVs are thought to contribute to multiple intercellular signaling pathways, including those involved in neurodegeneration. Although current antiretroviral therapy (ART) has reduced the severity of HIV-associated neurocognitive disorder (HAND), EVs released from latently infected cells may still drive residual neurotoxic effects. Datta’s group demonstrated that EVs derived from HIV-infected macrophages disrupted neural network formation, altered actin dynamics, and impaired mitochondrial function.

Next, Partha Chandra (Tulane University School of Medicine) delivered a talk titled “Circulating plasma extracellular vesicles indicate dysregulation of synaptic signaling in SHIV-infected rhesus macaques”. Using multiple experimental models, HIV-derived EVs were shown to induce mitochondrial hyperfusion in brain endothelial cells, potentially contributing to microvascular dysfunction and BBB leakage. Plasma-derived EVs from both SHIV-infected and uninfected rhesus macaques contained CNS cell-specific proteins. Proteomic analysis further revealed dysregulation of synaptic signaling pathways in the SHIV-infected animals.

The third speaker, Catherine DeMarino (NIH/NINDS), presented “HIV-1 RNA in extracellular vesicles is associated with neurocognitive outcomes”. Despite ART, viral RNA continues to be incorporated into EVs. Distinct transcriptional profiles were observed in CSF and blood, with at least one viral RNA detected in every CNS sample, indicating that the CNS remains a transcriptionally active reservoir. Importantly, EV-associated viral RNAs correlated with neurocognitive dysfunction in a cross-sectional longitudinal study.

The final speaker, Seema Singh (University of Nebraska Medical Center), discussed “Regulation of HIV Tat-mediated release of ferroptosis cargoes in microglia-derived extracellular vesicles involves microglial ferritinophagy”. HIV infection of microglia triggers the release of proinflammatory cytokines and other mediators that impact astrocyte and neuronal function. Singh’s work showed that microglia exposed to HIV Tat released EVs enriched in ferroptotic cargoes and exhibited dysregulated ferritinophagy. Notably, treatment with ferroptosis inhibitors reduced Tat-mediated release of these cargos, suggesting a mechanistic link between autophagy, EV release, and cargo composition.

This session was followed by an NIH grant writing workshop led by Fatah Kashanchi.

**Day 2 of the meeting:** The afternoon session on EVs and the CNS was moderated by Piul Rabbani (New York University) and Suvendra Bhattacharyya (University of Nebraska Medical Center).

The first speaker, Sarah Baker (Oregon Health and Science University), presented “The effect of Niemann Pick Disease Type C on extracellular vesicle concentration and cargo”. Neimann-Pick Disease Type C (NPC) shares pathological features with Alzheimer’s disease. NPC cell lines exhibit cholesterol accumulation in lysosomal compartments, leading to oxidative stress, impaired autophagy, and lipid accumulation. CSF samples from NPC patients showed higher EV concentrations compared with controls, with EVs enriched in Lamp1. Additionally, miRNAs associated with cholesterol homeostasis and autophagy were upregulated in NPC-CSF EVs.

Next, Julie Saugstad (Oregon Health and Science University) presented “Cerebrospinal fluid extracellular vesicle miRNAs identify synaptic alteration in human Alzheimer’s disease brain”. CSF miRNA expression is altered in Alzheimer’s disease (AD) and appears to be influenced by APOE genotype. Several miRNAs associated with synaptogenesis were increased in CSF EVs from AD patients. The study also demonstrated that the miRNA cargo of these EVs is affected by sex, genetics, and disease state.

Ashley Russell (Penn State Erie, The Behrend College) gave the next presentation, entitled “Myelin associated protein expression and extracellular vesicle composition are altered by endoplasmic reticulum stress in oligodendrocytes”. ER stress was shown to reduce the expression of key myelination proteins while increasing the expression of autophagy-related proteins. EVs released from ER-stressed cells exhibited increased expression of the autophagosomal protein, LC3B. Interestingly, a plate-based assay measuring autophagosome formation revealed significant decreases in ER-stressed cells. These findings highlight the need for further investigation into the crosstalk between ER stress, autophagy, and EV release and composition.

The last speaker of the session, Candice Brown (West Virginia University), presented “Experimental ischemic stroke in mice with an endothelial cell deletion of tissue-nonspecific alkaline phosphatase generates circulating extracellular vesicles that impair brain endothelial cell function”. Tissue-nonspecific alkaline phosphatase (TNAP) is expressed at the BBB, and its activity is decreased in brain microvessels following ischemic stroke. Mice with endothelial-specific TNAP deletion (Alpl^ECKO^) and their littermate controls (Alpl^fl/fl^) underwent ischemic stroke or sham surgeries. EVs were isolated from blood plasma, and human endothelial cells treated with stroke Alpl^ECKO^ EVs showed reduced viability and increased cytotoxicity. These effects were particularly pronounced with EVs derived from female Alpl^ECKO^ stroke mice.

The second afternoon session: EVs and Cancer, was moderated by Lucia Languino (Thomas Jefferson University).

This session began with a keynote presentation by Wei Guo (University of Pennsylvania) entitled “Extracellular vesicles in immune suppression and tumor progression”. Wei Guo highlighted that immune-suppressive EVs are present in the sera of patients with multiple cancer types, including metastatic melanoma, oral squamous cell carcinoma, and ovarian cancer. Small EVs (sEVs) carrying PDL1 were shown to inhibit T cell function *in vitro* and can also induce apoptosis of CD8^+^ T cells. In addition to PDL1, adhesion molecules such as ICAM1 were co-expressed on sEVs, facilitating their interaction with T cells. Furthermore, increased expression of LFA1 on activated T cells enhances their association with sEVs. Chimeric antigen receptor (CAR) T cells were noted to be particularly susceptible to tumor-derived sEVs carrying both PDL1 and target tumor antigens, which may promote preferential binding to CAR T cells and contribute to CAR T cell exhaustion. Wei Guo also demonstrated that phosphorylated HRS, a member of the ESCRT complex, mediates the loading of PDL1 into sEVs.

Lance Liotta (George Mason University) followed with a presentation entitled “Mitophagy-associated EVs mediate cancer cell survival by exporting damaged mitochondria components bound to tumor suppressor proteins, offering new mechanistic strategies for cancer prevention and therapy”. He explained that mitogenesis and mitophagy are energy-demanding processes critical for normal cellular function. PINK1 triggers the sequestration and removal of damaged mitochondria, and after acute oxidative stress, full-length PINK1-positive EVs containing damaged mitochondrial components are observed. This process may support cancer cell survival, as tumor suppressor proteins such as p53 and merlin are also packaged in these EVs. The export of p53 via EVs can increase genetic instability in cancer cells and drive cancer progression.

Next, Nykia Walker (University of Maryland, Baltimore County) presented “Tumor-derived extracellular vesicles systemically reprogram naïve mammary stroma for breast cancer metastasis through Let-7 miR suppression”. This unpublished work focused on using bioinformatics to identify invasive signaling pathways and to explore the biological and clinical significance of tumor-derived EV-regulated gene sets in promoting tumor cell invasion.

Camila Bach (Thomas Jefferson University) then presented “A novel sialylation pathway mediated by extracellular vesicles in aggressive prostate cancer”. She discussed how myeloid-derived suppressor cells contribute to immune suppression and prostate cancer immune escape, shaping tumor progression and therapy resistance. Altered glycosylation on T cell surfaces is a hallmark of cancer and correlates with poor clinical outcomes and immune suppression. Unpublished data suggest that sEVs may play an important role in modulating cellular glycosylation within the cancer context.

The session concluded with Theresa Whiteside (University of Pittsburgh), who presented “Melanoma-derived exosomes induce intrinsic apoptosis of activated T cells by early transcriptional activation of cellular stress response genes”. Whiteside reported that melanoma-derived EVs (MTEX) carry a variety of proteins and can be taken up by T cells, inducing mitochondrial damage and triggering the intrinsic apoptosis pathway. The effects of MTEX on mitochondrial integrity were shown to be both time- and concentration-dependent. Gene expression analyses revealed that multiple pathways may be involved in mediating these effects, warranting further investigation.

**Day 3 of the meeting:** The Saturday morning session, entitled “EVs and Technology”, was moderated by Heather Branscome (American Tissue Culture Collection) and Daniel Chiu (University of Washington), who presented a study titled “High-resolution analysis of single extracellular vesicles and particles with digital flow cytometry and super-resolution imaging”. They described a novel high-throughput, high-resolution, multi-parametric technology for analyzing EVPs using an innovative digital flow cytometer (dFC) from Pangnostics. This microfluidics-based instrument offers a high signal-to-noise ratio and enables quantification of the number of molecules of any protein on the EVP surface or within internal cargo. Additionally, the instrument features sorting capabilities, allowing 10,000 EVPs to be sorted with 98% purity.

The next speaker, Piul Rabbani, presented “Topically delivered exosomes modulate innate immune components for diabetic wound closure”. He noted that the pathology of chronic wounds is not adequately addressed in routine clinical care, resulting in a significant burden on the healthcare system. sEVs derived from allogenic multipotent stromal cells (MSCs) can facilitate tissue repair. These EVs are incorporated into Q fibers (ExoQ), which can then be applied to wounds to enable sustained delivery of sEVs. A single dose of ExoQ was shown to accelerate diabetic wound closure by nearly 50%. Interestingly, macrophages at the wound site also appear to play an important role in healing, warranting further investigation.

Next, Matt Kremer (Univercells Technologies) presented “Scale-X bioreactors for adherent cell culture applications”. He highlighted a fixed-bed bioreactor that uses layers of non-woven, medical-grade polyester, allowing cells to adhere and proliferate. This design supports high cell density, low shear stress, and linear scalability, enabling increased cell numbers and correspondingly higher EV output.

The session concluded with a roundtable discussion featuring several NIH program officers, who discussed various EV-focused funding opportunities.

The final session of the meeting, EVs and Technology 2, was moderated by Julie Saugstad (Oregon Health & Science University) and Nicole Noren Hooten (NIH/NIA).

Javaria Munir (University of Nebraska - Lincoln) kicked off the session with a presentation entitled “Immortalized mammary alveolar cells (MAC-T) as a tool for designing organ-specific targeting exosomes via genetics and click chemistry approach”. The BBB is highly restrictive, making drug delivery to the brain particularly challenging. EVs may serve as biological nanocarriers; for example, milk-derived EVs have been shown to accumulate in the brain during suckling, and bovine milk EVs have been detected in mouse brains after oral administration. Because these EVs are resistant to degradation in the gastrointestinal tract and do not trigger cytokine storms in recipient cells, they hold potential as drug delivery tools. Furthermore, bovine milk EVs decorated with a brain-homing signal, ApoE, showed enhanced bioavailability and brain accumulation.

Next, Sebastian Molnar (George Mason University) presented “EV isolation methods identify distinct HIV-1 particles released from chronically infected T cells and PBMCs”. Extracellular particles, including both vesicular and non-vesicular forms, are highly heterogeneous in size. Infectivity assays demonstrated that HIV is present in fractions containing particles of varying sizes, each with distinct characteristics.

Michelle Pleet (NIH/NINDS) then discussed “A workflow for the identification and capture of EV subsets in CNS diseases and cancer”. Cells and EVs obtained via lumbar puncture can be separated and immunophenotyped to enable a comprehensive study of CSF. While no differences were observed in CSF EV size or concentration among healthy volunteers, multiple sclerosis (MS) patients, and patients with viral-mediated neurological disease resembling MS, distinct protein markers were detected on EVs captured using Nanovials. These hydrogel beads, previously used for single-cell capture, have a cup-like structure containing a capture antibody and were shown to effectively capture EV subtypes. They enabled the identification of disease-associated EV subtypes in CSF and successfully distinguished cancer-specific markers between glioblastoma and colon cancer EVs.

Duaa Dakhlallah (West Virginia University) presented “Targeting the epigenetic modifications in vascular endothelial cells during sepsis via circulating extracellular vesicles”. Sepsis is a leading cause of death in non-coronary ICUs, yet rapid diagnosis and treatment can improve outcomes. Epigenetic modifications during critical phases of sepsis arise from host-pathogen interactions, acute inflammation, and immune suppression. DNA methylation is regulated by DNMT3a and DNMT3b (*de novo* DNA methylation) and DNMT1 (maintenance). Infection can modulate DNA methylation, and patients in septic shock show elevated circulating EV levels with increased DNMT1 and DNMT3a activity, which can transfer to recipient cells. These EVs also downregulate proinflammatory cytokines at both transcriptional and translational levels.

Nicole Noren Hooten presented “Exploring extracellular vesicle mitochondrial DNA levels with chronic kidney disease and mitochondrial haplogroup in obese individuals”. Chronic kidney disease (CKD) affects over 35 million Americans, with higher prevalence in African Americans. EV-encapsulated mitochondrial DNA (mtDNA) isolated from plasma revealed that African Americans who develop CKD exhibit lower mtDNA levels compared to controls or white Americans with CKD. mtDNA haplogroups influence mitochondrial function, and the African haplogroup showed lower EV mtDNA levels. Longitudinal EV mtDNA levels were found to depend on haplogroup, CKD status, age, and sex.

The final speaker, Jason Lowery (Beckman), discussed the experimental workflow for EV separation and characterization, highlighting ultracentrifugation in EV research and introducing the new FLEX Family (CytoFLEX) analyzer and sorter.

The meeting concluded with closing remarks from members of the ASIC organizing committee, and awards were presented for the top three oral and poster presentations. The oral presentation winners were Catherine DeMarino (First place), Lillian Skeiky (Second place) and Camila Bach (Third place). Poster presentation winners were Ahana Byne (First place), Sebastian Molnar (Second place), and Anastasia Williams (Third place).

ASIC President Fatah Kashanchi (George Mason University) expressed gratitude to the Organizing Committee for their efforts in assembling the meeting, with special recognition for Gwen Cox for her consistent dedication to ensuring smooth and efficient execution.

Researchers at this meeting shared major advances in EV research across diverse biological systems and disease contexts. Presentations highlighted the growing translational potential of EVs as diagnostic and therapeutic tools, and notable progress was reported in elucidating mechanisms underlying EV biogenesis, cargo selection, and intercellular communication. Across sessions, adoption of innovative technologies signaled a shift toward more precise and sophisticated EV research, underscoring the importance of standardization, technological advancement, and cross-disciplinary collaboration.

Several talks offered particularly novel and impactful insights. Alissa Weaver’s keynote highlighted a VAP-A-dependent pathway for RNA loading into small, dense EVs. Wei Guo’s keynote described how tumor-derived sEVs suppress immune responses by inhibiting T cell function. The EVs and CNS session featured compelling findings from Prasun Datta, Partha Chandra, Catherine DeMarino, and Seema Singh, who demonstrated that EVs contribute to neurocognitive dysfunction by carrying viral RNA, disrupting mitochondrial and synaptic function, and containing ferroptosis-related cargo, linking EVs to viral persistence and neuroinflammation. Lance Liotta presented evidence that EVs can export damaged mitochondria and tumor suppressor proteins such as p53, potentially promoting cancer progression. Lillian Skeiky shared clinical data linking EV-associated cytokines to sleep disruption following total sleep deprivation, a novel and understudied area of EV biology. Candice Brown reported that post-ischemic stroke, EVs from mice lacking brain endothelial TNAP impaired endothelial cell viability, with stronger effects from EVs derived from female mice. Javaria Munir demonstrated the potential of milk-derived EVs for brain-targeted drug delivery, and Piul Rabbani introduced a novel EV-based wound healing strategy using Q fiber-embedded vesicles for diabetic wound care.

## DECLARATIONS

### Copyright

© The Author(s) 2025.

